# m^6^A RNA methylation regulator-based signature for prognostic prediction and its potential immunological role in uterine corpus endometrial carcinoma

**DOI:** 10.1186/s12885-022-10490-x

**Published:** 2022-12-29

**Authors:** Fang Fang, Peipei Wang, Haoyu Huang, Mingquan Ye, Xiaocen Liu, Qingqing Li

**Affiliations:** 1 Department of Laboratory Medicine, Yijishan Hospital, the First Affiliated Hospital of Wannan Medical College, Wuhu, 241001 China; 2 Anhui Province Clinical Research Center for Critical Respiratory Medicine, Yijishan Hospital, the First Affiliated Hospital of Wannan Medical College, Wuhu, 241001 China; 3 School of Medical Information, Wannan Medical College, Wuhu, 241001 China; 4 Research Center of Health Big Data Mining and Applications, Wannan Medical College, Wuhu, 241001 China; 5 Department of Nuclear medicine, Yijishan Hospital, the First Affiliated Hospital of Wannan Medical College, Wuhu, 241001 China

**Keywords:** UCEC, m6A RNA methlation, Prognostic prediction, Gene signature

## Abstract

**Background:**

Uterine corpus endometrial carcinoma (UCEC) is the most common female pelvic malignancy worldwide. N6-methyladenosine (m^6^A) plays an important role in various cellular responses, especially in cancer progression. However, the correlation between prognostic UCEC and m^6^A RNA methylation regulators remains unclear.

**Methods:**

We used The Cancer Genome Atlas (TCGA) to provide a gene signature that could improve the prognostic evaluation of UCEC patients according to the distinct genetic trait of m^6^A RNA methylation regulators from a bioinformatics perspective. After comparing UCEC subgroups with different genetic profiles of m^6^A regulators, we identified 71 differentially expressed genes associated with overall survival (OS) and generated a nine-gene signature through least absolute shrinkage and selection operator (LASSO) Cox regression analysis. Finally, we used in vitro and in vivo tumor cell experiments as well as the immune correlation analysis to verify the function of each gene in the proposed gene signature.

**Results:**

Time-dependent receiver operating characteristic (ROC) curves revealed that the proposed gene signature could predict the outcome of UCEC patients accurately. We found that *CDKN2A* mainly acted from the perspective of tumor cells, while *COL4A4*, *PXDN*, *TIGIT*, *CHODL*, *LMO3*, *KCNJ12*, *L1CAM,* and *EPHB1* might play a role in UCEC from an immunological point of view.

**Conclusions:**

From an epigenetics perspective, the m6A RNA methylation regulator-based gene signature can predict the prognosis of UCEC patients and immune therapeutic efficacy.

**Supplementary Information:**

The online version contains supplementary material available at 10.1186/s12885-022-10490-x.

## Background

Uterine corpus cancer is the most common female pelvic malignancy (with an estimated 65,620 new cases) and ranks as the third leading cause of gynecological cancer-related death (estimated to be 12,940, following breast cancer at 43,600 and ovarian cancer at 22,950) [1]. Endometrial carcinomas account for most of these cases and encompass more than 83% of uterine corpus cancers [2]. The incidence of uterine corpus endometrial carcinoma (UCEC) is increasing due to prolonged life expectancy and early diagnosis. However, most patients face a risk of recurrence after surgery or multi-therapies, leading to a poor prognosis. Thus, it is urgent to identify novel therapeutic targets and prognostic biomarkers to provide personalized treatment for UCEC. In recent years, comprehensive studies of new prognostic signatures based on epigenetic characteristics have been elucidated [3, 4]. Cancer cell-intrinsic epigenetic alterations have been associated with carcinogenesis and tumor progression. However, few studies have reported prognostic signatures based on epigenetics in UCEC, possibly because epigenetics is not widely studied in UCEC.

N^6^-methyladenosine (m^6^A) methylation modification refers to the addition of a methylated group to the N6 position of adenosine, which is the most abundant internal modification of eukaryotic mRNA and lncRNA relative to N^1^-methyladenosine (m^1^A) and 5-methylcytosine (m^5^C) [5]. Modification by m^6^A is a dynamic and reversible process, which is regulated by m^6^A methyltransferases, m^6^A demethylases, and m^6^A-binding proteins, i.e., writers, erasers, and readers (WERs). In the process, methylation and demethylation can occur simultaneously. The installation of m^6^A can affect alternative pre-mRNA splicing, 3′-end processing, mature mRNA nuclear export, and mRNA degradation. Therefore, it can affect gene expression at the transcriptional level in several ways and is directly linked with protein translation [6, 7]. Methylation of mRNA m^6^A is essential for different physiological activities, including human diseases, by impacting gene transcription [8]. The m^6^A regulators are closely associated with the malignancy in various cancers because m^6^A regulators can directly affect the expression of oncogenes or tumor suppressors, and certain m^6^A regulators serve as prognosis biomarkers for certain cancers [9, 10]. The m^6^A methylation in nearly 70% of endometrial tumors is reduced, thus promoting the proliferation and oncogenicity of endometrial cancer cells [11]. However, the correlation between UCEC prognostics and m^6^A RNA methylation status remains unknown.

Recently, numerous studies have found that m^6^A modification can participate in regulating the composition of the tumor microenvironment (TME) infiltrating cells to modulate the immune response [12]. For example, RNA methyltransferase METTL3-mediated m^6^A methylation can promote dendritic cell (DC) activation and antigen-presenting cells (APC)-based T cell response [13]. YTHDF1 depletion can enhance CD8^+^ T cell-related response and immuno-therapeutic efficacy of PD-L1 checkpoint blockade [14]. Therefore, m^6^A modification status can modulate the efficiency of the antitumor immune response, the immune-therapeutic efficacy, and the overall survival (OS). Since epigenetic reprogramming is more likely manipulated by targeting epigenetic modifiers, epigenetic biomarkers may offer additional advantages [15]. However, until now, the relevance between m^6^A regulators and TME in UCEC remains unclear. Thus, an inquiry into m^6^A methylation may provide more possibilities for predicting immune therapeutic efficacy and the prognosis of UCEC.

With the rapid growth of high throughput sequencing technologies, there is an opportunity to obtain an intact picture of the mutation and transcription landscape of most tumors with even more clarity. Herein, we explored the landscape of m^6^A regulators in a UCEC cohort from The Cancer Genome Atlas (TCGA) database. We subgrouped UCEC patients according to their genetic alterations in m^6^A regulators because alteration in even one m^6^A regulator could lead to a dysfunction of m^6^A RNA modification, resulting in dysregulation of gene expression [16]. Then, we compared the molecular, clinical, and immunological characteristics between groups with distinctions of m^6^A regulators using a least absolute shrinkage and selection operator (LASSO) Cox regression analysis. A nine-gene prognostic mRNA signature was generated to predict the OS of UCEC patients. Finally, the function of each gene in the proposed gene signature was verified using in vitro and in vivo tumor cell experiments as well as the immune correlation analysis. The result shows that *CDKN2A* mainly acts from the perspective of tumor cells while the remaining genes mainly act by regulating the immune response. Therefore, the m^6^A RNA methylation regulator-based signature can provide a robust and stable prognostic tool and help probe the potential therapeutic target for UCEC treatment from the perspective of epigenetics.

## Methods

### Datasets for analysis

All paired clinical data and transcript profiles of UCEC samples were obtained and trimmed from the TCGA Data Portal by R package “GDCRNATools” and all the somatic mutations were available from the mutation annotation format (MAF) file on the TCGA website directly. A total of 530 UCEC samples were retained, and only patients with somatic mutation data and complete follow-up clinical information were screened for subsequent studies. Based on the R package “caret”, TCGA UCEC profiles were randomly separated into two parts: 75% was the training set, and 25% was the testing set. All methods were carried out in accordance with relevant guidelines.

### Visualization of genetic alterations in m^6^A regulators

A total of 20 representative m^6^A regulators, including ten readers, eight writers, and two erasers, were selected from recently published studies. Before further analysis, all gene symbols of these m^6^A regulators were converted into HUGO Gene Nomenclature Committee (HGNC) symbols by manual curation from Ensembl (http://asia.ensembl.org/index.html). The R package “maftools” was used to summarize, analyze, and visualize the somatic mutations [17]. The summary of the MAF file was visualized as a waterfall plot, showing the number of variants in each sample. The variant allele frequency of gene mutations, shown as a boxplot, was defined as the reading of variants divided by the total reading.

### Recognition of m^6^A-related differentially expressed genes (DEGs)

Alterations in m^6^A regulatory genes were screened in each UCEC sample as above. Subsequently, one cohort was separated into two subgroups: one group with m^6^A alterations and one without m^6^A alterations. The R package “Deseq2” was adopted to generate the DEGs, which depended upon a negative binomial distribution between the two groups. *P* < 0.05 and |log_2_ Fold-change| ≥ 1 were taken as significance criteria for DEGs. The R package “pheatmap” was adopted to conduct unsupervised learning clustering and plot the heatmap.

### Immune cell composition estimation and gene ontology (GO) enrichment analysis

Tissue-infiltrating immune and other stromal subpopulation abundances based on mRNA expression were estimated by the MCP-counter method implemented by the R package “MCPcounter”. The immune cell composition estimation results were visualized using the R package “vioplot” in a violin plot. The online web server g:Profiler (https://biit.cs.ut.ee/gprofiler/gost) was used to perform an ordered GO enrichment analysis, where genes are in the order of decreasing importance [18]. Then, pathway enrichment was visualized and interpreted with the Cytoscape (V3.8.0) desktop application and the “EnrichmentMap Pipeline Collection” plug-in.

### Modeling of m^6^A-related signatures for forecasting OS

The R package “glmnet” was used to conduct the LASSO Cox regression model via penalized maximum likelihood. The optimal cut-off point for distinguishing between high- and low-risk rating groups was determined using the R package “survminer” based on log-rank statistics. The m^6^A score formula was generated, and the risk score of each patient was calculated as follows:

$$\boldsymbol{Risk}\ \boldsymbol{score}=\sum_{\boldsymbol{i}=\textbf{1}}^{\boldsymbol{n}}{\boldsymbol{Coef}}_{\boldsymbol{i}}\times {\boldsymbol{Exp}}_{\boldsymbol{i}}$$

where *Coef*_*i*_ means the coefficient and *Exp*_*i*_ is the expression of each m^6^A-related mRNA. Receiver operating characteristic (ROC) curves and area under the ROC curve (AUC) were used to quantify the sensitivity and specificity of the prognostic signature through the R package “timeROC”.

### Tools for further analysis of the m^6^A signature

GETPIA 2 (http://gepia2.cancer-pku.cn/) [19], an enhanced web server for large-scale expression profiling and interactive analysis, was used to determine the relevance of the m^6^A signature gene expression and clinical outcome across various cancer types. CIBERSORT (https://cibersort.stanford.edu/) [20], an analytical tool to estimate the abundances of member cell types, was used to calculate the correlation between the indicated gene and tumor-infiltrating immune cells. RMBase v2.0 (https://rna.sysu.edu.cn/rmbase/) [21], a comprehensive database to integrate epi-transcriptome sequencing data for exploring post-transcriptionally modifications of RNAs, was used to decipher different RNA modifications of the signature, including m^6^A, N1-Methyladenosines (m^1^A), pseudouridine (Ψ) modifications, 5-methylcytosine (m^5^C) modifications, 2′-O-methylations (2′-O-Me). M6A2Target (http://m6a2target.canceromics.org/) [22], a comprehensive database for the target gene of WERs of m^6^A modification, was used to illustrate the correlation between WERs and the m^6^A signature.

### Cell transfection to obtain knockdown cell lines

The lentivirus construction used to knockdown *COL4A4*, *PXDN*, *CDKN2A*, *TIGIT*, *CHODL*, *LMO3*, *KCNJ12*, *L1CAM,* and *EPHB1*was purchased from Genepharma (Shanghai, China). UCEC cell line Ishikawa was plated in 6-well microplates at 40–50% confluence and then infected with the above 9 lentiviruses (termed as shCOL4A4, shPXDN, shCDKN2A, shTIGIT, shCHODL, shLMO3, shKCNJ12, shL1CAM, and shEPHB1), or control (termed as shCtrl), respectively. Stable transduction pools were generated by puromycin selection for 2 weeks. The above cell transfection protocol was based on the manufacturer’s instructions.

### Western blot assay

Western blot assay was performed as previously reported [23]. In brief, the proteins from Ishikawa cells were exacted with RIPA lysis buffer (Sigma-Aldrich, St Louis, MO). The concentration of proteins was measured with a BCA assay kit (Bio-Rad Laboratories, Hercules, CA, USA). The 10% SDS-PAGE isolated protein was transferred to a 0.22-μm nitrocellulose (NC) membrane (GE Healthcare, Piscataway, NJ, USA). The membranes were blocked at room temperature with 5% non-fat milk for 2 h. Then, the membranes were incubated in specific primary antibodies including COL4A4 (1:1000, MBS2032561, MyBioSource, San Diego, CA, USA), PXDN(1:1000, sc-293,408, Santa Cruz, CA, USA), CDKN2A(1:1000, ab270058, Abcam, Cambridge, MA, USA), TIGIT(1:1000, ab243903, Abcam, Cambridge, MA, USA), CHODL(1:1000, ab236742, Abcam, Cambridge, MA, USA), LMO3(1:1000, ab230490, Abcam, Cambridge, MA, USA), KCNJ12(1:1000, PA5–68685, Invitrogen, Carlsbad, CA, USA), L1CAM(1:1000, ab24345, Abcam, Cambridge, MA, USA), EPHB1(1:1000, ab129103, Abcam, Cambridge, MA, USA) and β-Actin (1:3000, A1978, Sigma, Victoria, BC, Canada) at 4 °C overnight. The membranes were washed with 0.1% TBST three times for 5 min, and then incubated with anti-mouse or anti-rabbit horseradish peroxidase-conjugated secondary antibody (Cell Signaling Technology, Danvers, MA, USA) for 2 h, and washed with 0.1% TBST three times for 5 min each. Chemiluminescent ECL Plus reagents (Pierce, USA) were added to visualize the reaction products. The membranes were scanned with Tanon 5200 (Tanon, Shanghai, PR China). The band intensity was measured by densitometry using the Quantity One Software (Tanon, Shanghai, PR China). The protein levels were normalized with that of β-actin. All experiments were repeated in triplicate, and the representative results were shown.

### MTT, Colony formation, Transwell, and wound healing assays

**MTT,** Colony formation and Transwell assays were performed as described previously [23]. Cell migration was also performed with a wound healing assay. Ishikawa cells transfected with the shCOL4A4, shPXDN, shCDKN2A, shTIGIT, shCHODL, shLMO3, shKCNJ12, shL1CAM, shEPHB1 and shCtrl were seeded in 6-well microplates, and scratches were generated using micropipette tips when 90% confluence was reached. Cells were washed 3 times using sterile PBS to wash off non-adherent cells generated by the scratch, and a fresh serum-free medium was replaced to continue culturing the cells. The wound status was observed at 0 h and 72 h after scratching with an X71 inverted microscope. The means of intercellular distances were calculated using the ImageJ software. All experiments were duplicated thrice.

### Xenografted tumor model

Four to 6 weeks old (average weight: 15 g) BALB/c nude mice (male/female ratio 1:1) were obtained from the Shanghai Institute of Materia Medica, Chinese Academy of Science, and maintained under specific pathogen-free conditions. No statistical method was applied for the sample size estimation for the animal study. Each experimental group had enrolled 4 nude mice in an unrandomized manner to ensure the precision of the results. The experimental protocol was approved by the Wannan Medical College Animal Experimental Ethics Committee and reporting of these experiments complied with the ARRIVE (Animal Research: Reporting of In Vivo Experiments) guidelines. The cells (2 × 10^6^ Ishikawa -shCDKN2A cells and Ishikawa-shCtrl cells) were injected subcutaneously into the right dorsal flank. The tumor sizes were measured using a Vernier caliper every day when the tumors were readily visualized. Tumor formation in nude mice was observed by measuring the tumor volume calculated by the following formula: volume = (length × width^2)/2. On day 35, animals were euthanized, and tumors were excised and weighed. The exclusion criteria of animal experiments are that the bodyweight of the mouse was statistically significantly changed compared to the others. The xenograft tumor was festered seriously and influenced the measurement of tumor volume.

### Statistical analysis

Correlation analysis was conducted using the Pearson correlation method. The chi-square test was applied to compare the clinical-pathological features among different groups. The Mann-Whitney *U* test was adopted to compare the difference between genetic patterns. All statistical analyses were performed using R software (version 3.6.3) in the RStudio program (version 1.3.1073).

## Results

### Landscape of genetic alterations in m^6^A regulators in TCGA UCEC patients

**Fig. 1:**
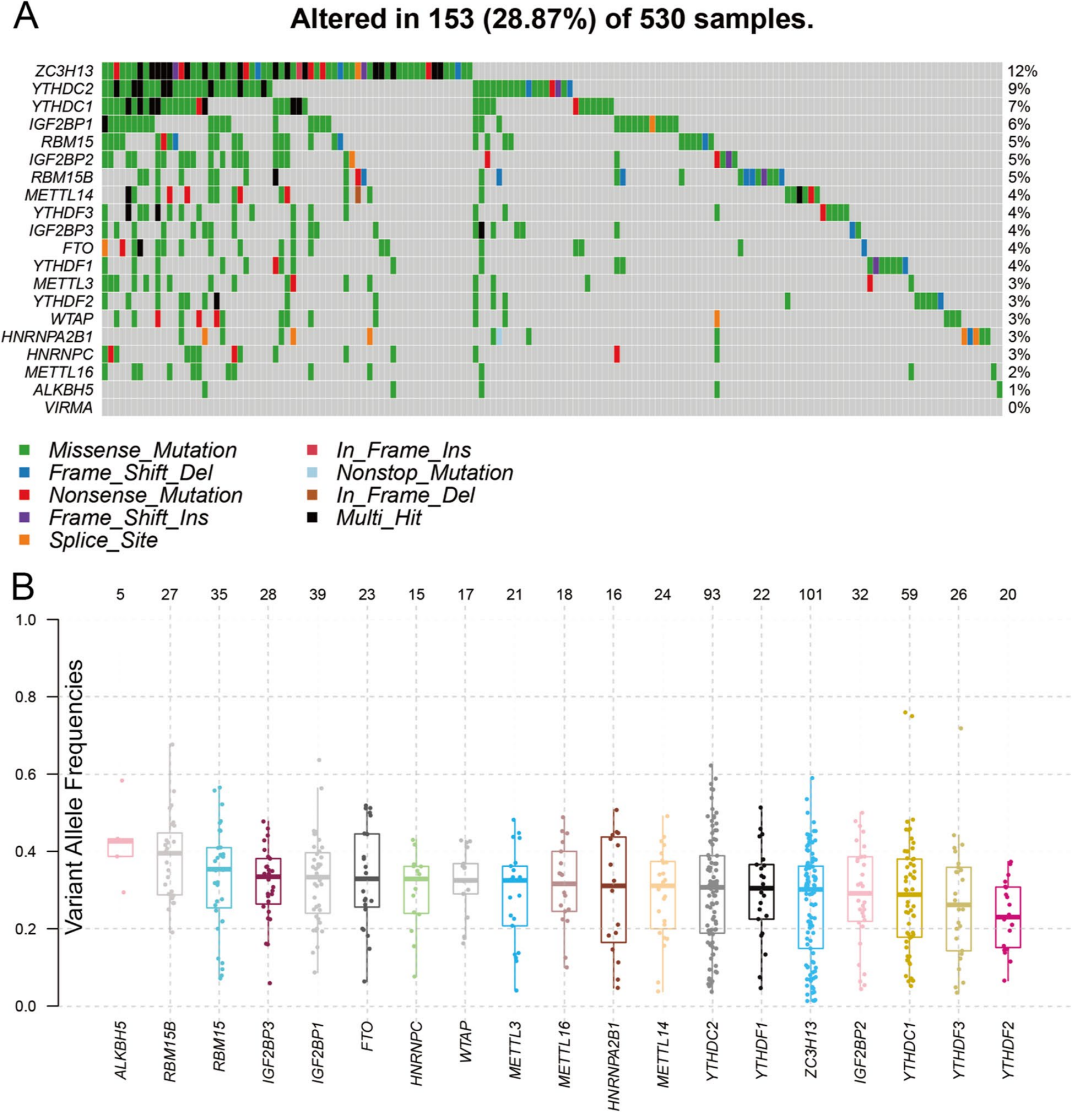
Genetic alterations in m^6^A regulators in uterine corpus endometrial carcinoma (UCEC). **A** The waterfall plot shows the number of altered m^6^A regulators in each UCEC sample. **B** The boxplot shows the variant allele frequency of gene mutations, defined as the reading of variant reads separated by the total reading

Among 530 TCGA UCEC cases that were analyzed, 153 samples from all cases had a genetic alteration in the m^6^A regulator with a frequency of 28.9%, and missense mutations had a leading role. The frequency of genetic alteration in the m^6^A writer gene *ZC3H13* (11.9%) was the highest, followed by *YTHDC2* (8.7%), *YTHDC1* (6.6%), *IGF2BP1* (6.0%), and *RBM15* (5.3%) (Fig. 1A; Table 1). Figure 1B shows the variant allele frequency of gene mutations, which refers to the ratio between the number of mutants and wild-type DNA copies, which estimates the clonal status of mutated genes (Fig. 1B). We searched for alterations in m^6^A regulatory genes in each TCGA UCEC sample. Then, TCGA cohorts were separated into two subgroups: one group with m^6^A regulator mutations and one group without m^6^A regulator mutations. Considering that an alteration in only one m^6^A regulator can lead to a dysfunction of m^6^A RNA modification, these two groups can represent two scenarios (a normal status and an abnormal one) of the m^6^A level.

**Table 1:** Different genetic alteration patterns of m^6^A regulators in UCEC samples (*n* = 530)

***Gene***	***Genetic Alteration***	***Altered Samples***	***% in Total***
**Writers**			
METTL3	21	18	3.4
METTL14	24	21	3.96
METTL16	18	13	2.45
WTAP	17	17	3.21
RBM15	35	28	5.28
RBM15B	27	24	4.53
VIRMA	0	0	0
ZC3H13	101	63	11.89
**Erasers**			
FTO	23	19	3.58
ALKBH5	5	5	0.94
**Readers**			
YTHDC1	59	35	6.6
YTHDC2	93	46	8.68
YTHDF1	22	19	3.58
YTHDF2	20	18	3.4
YTHDF3	26	21	3.96
IGF2BP1	39	32	6.04
IGF2BP2	32	25	4.72
IGF2BP3	28	20	3.77
HNRNPC	15	14	2.64
HNRNPA2B1	16	15	2.83

### Relationship among genetic alterations, m^6^A gene expression, and clinical phenotypes

We explored the relevance of genetic alterations, m6A gene expression, and the clinical phenotypes of UCEC patients. The results demonstrated that alterations in m^6^A regulators were only significantly related to age, not clinical stage and race. In Table 2, values in bold with an asterisk indicate a statistically significant difference (*P* < 0.05) using the chi-square test and demonstrate that genetic alterations in m^6^A regulators are more likely to occur in UCEC patients younger than 60 years of age. Supplementary Fig. 1 shows the decreasing trend of mRNA levels of *ALKBH5*, *FTO*, *METTL16*, and *RBM15* by the Mann-Whitney *U* test. It seems that genetic alterations, especially single-nucleotide substitutions by missense mutations, may not impair most mRNA expression. However, a single nucleotide alteration in the DNA sequence of a gene can produce the wrong amino acid, thus affecting the three-dimensional structure and biological function of an m^6^A regulator protein.

**Table 2:** Clinical phenotypes between UCEC patients with or without mutation of m^6^A regulators

***Variable***	***Without Mutation***	***With Mutation***	***chi-square value***	***P value***
Age				
< =60	128	73	9.578	0.002*
> 60	247	77		
Race				
White	262	99	4.393	0.222
Black	78	28		
Asian	11	9		
Others	24	14		
Clinical Stage				
Stage I	231	96	0.8286	0.8426
Stage II	35	16		
Stage III	89	31		
Stage IV	20	7		

### GO analysis for differentially expressed mRNAs

**Fig. 2:**
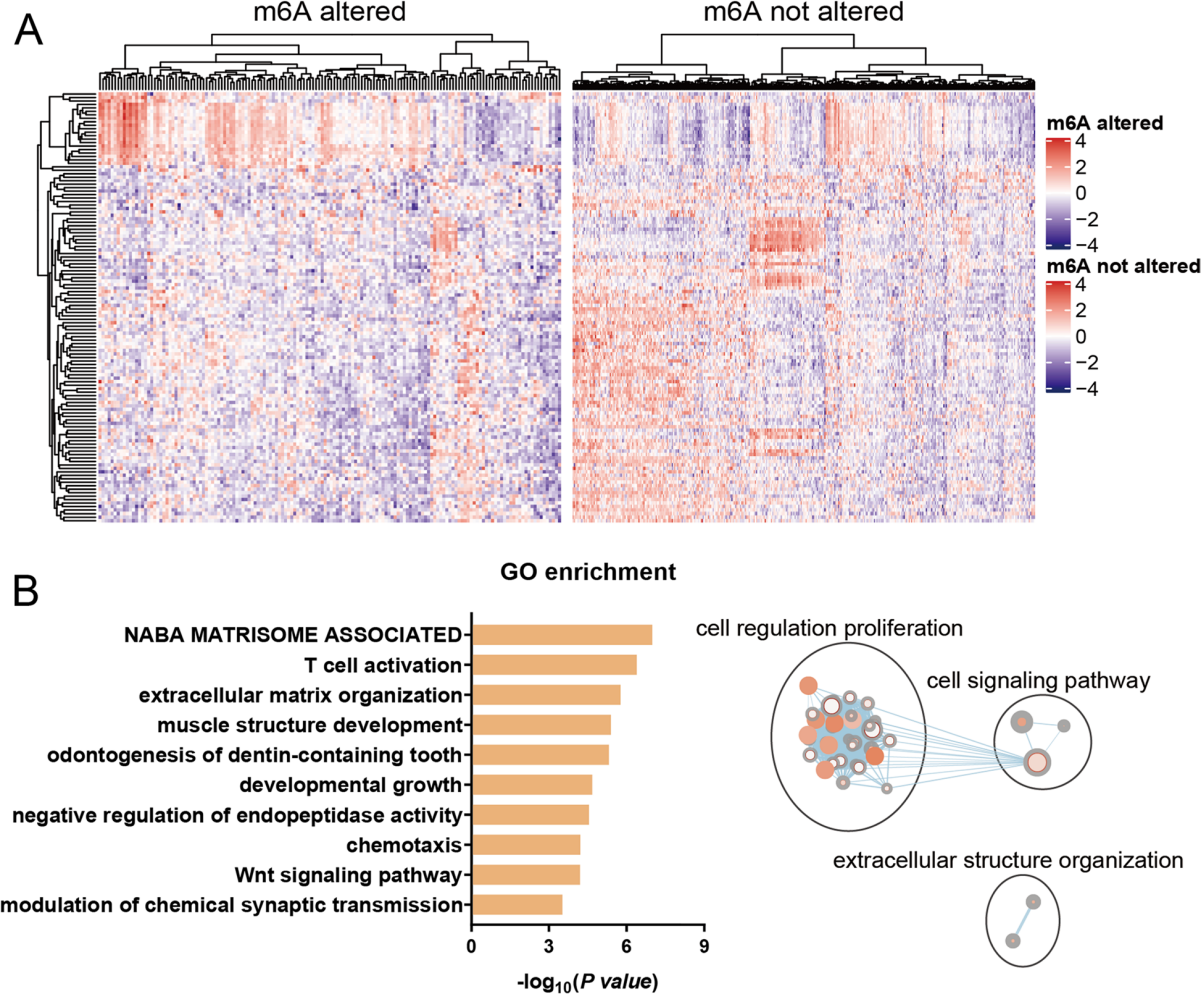
The m^6^A-related differences between the group with genetic alterations in the m^6^A regulator and the group without genetic alterations in the m^6^A regulator. **A** The heatmap shows that 124 mRNAs are differentially expressed between the two groups. **B** Gene Ontology (GO) enrichment of the differentially expressed mRNAs

A DEG analysis was conducted to recognize distinct patterns between subgroups with these two different m^6^A profiles. A total of 124 mRNAs passed the threshold screening. As shown in the heatmap (Fig. 2A), 23 up-regulated genes and 101 down-regulated genes are ranked by Pearson distance, indicating that m^6^A regulator-related genetic alterations can decrease most gene expression. GO analyses were conducted on these 124 genes. The results show that these differentially expressed mRNAs are mainly enriched in biological processes associated with T cell activation. A word cloud analysis shows that cell, regulation, and proliferation are the most frequent words enriched in GO terms (Fig. 2B). Therefore, m^6^A modification can affect the occurrence and development of tumors.

### Relationship between genetic alterations and immunological characteristics

**Fig. 3:**
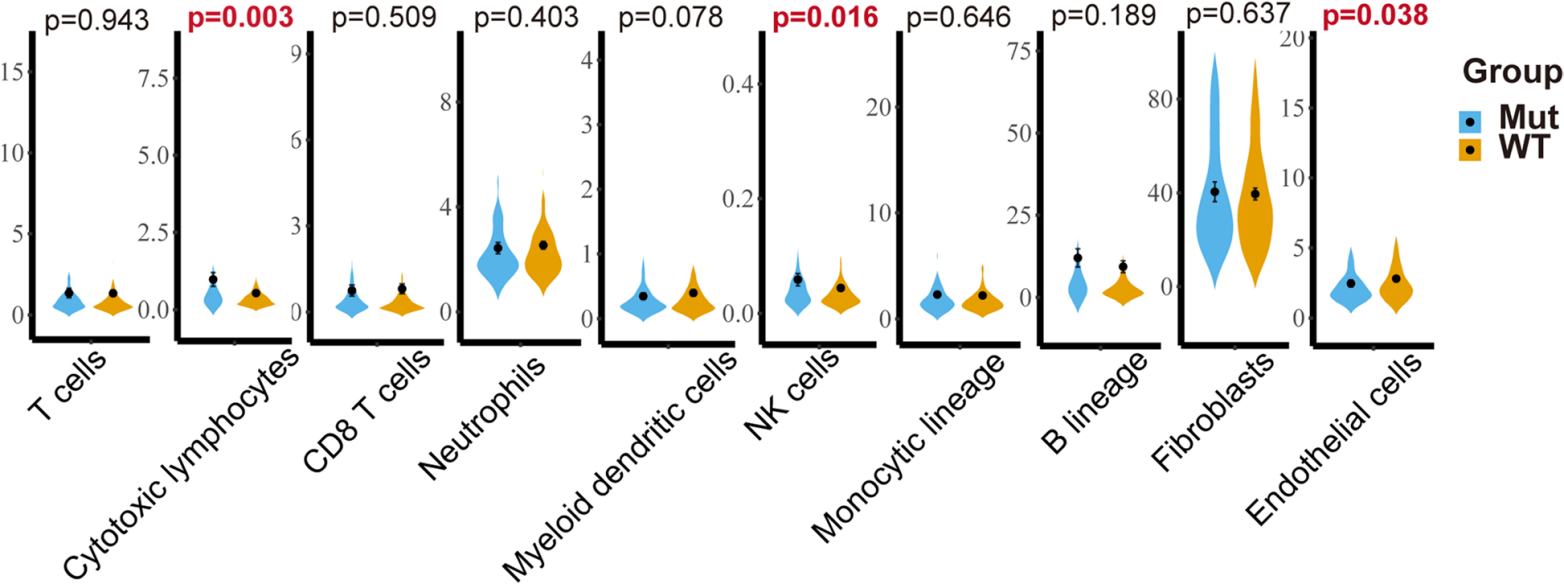
Immunological characteristics between UCEC patients with or without mutations in m^6^A regulators. Significant results are marked in red

Considering the potential roles of T cells above, the tissue-infiltrating immune and other stromal subpopulations abundances in UCEC groups with distinct m^6^A regulator traits were estimated to further explore the immunological role of m^6^A modification. The results are shown in the violin plot in Fig. 3, suggesting that cytotoxic lymphocytes and natural killer (NK) cells both can kill target cells and exhibit higher proportions in the UCEC group with alterations in the m^6^A regulator (Fig. 3). The results are consistent with the GO enrichment results above. In contrast, endothelial cells have the opposite role. There were no significant inter-group differences for other types of immune cells. These results show that distinct levels of m^6^A can lead to differential immune cell composition, thereby influencing the immune responses.

### Generation of m^6^A gene signature from the training set

**Fig. 4:**
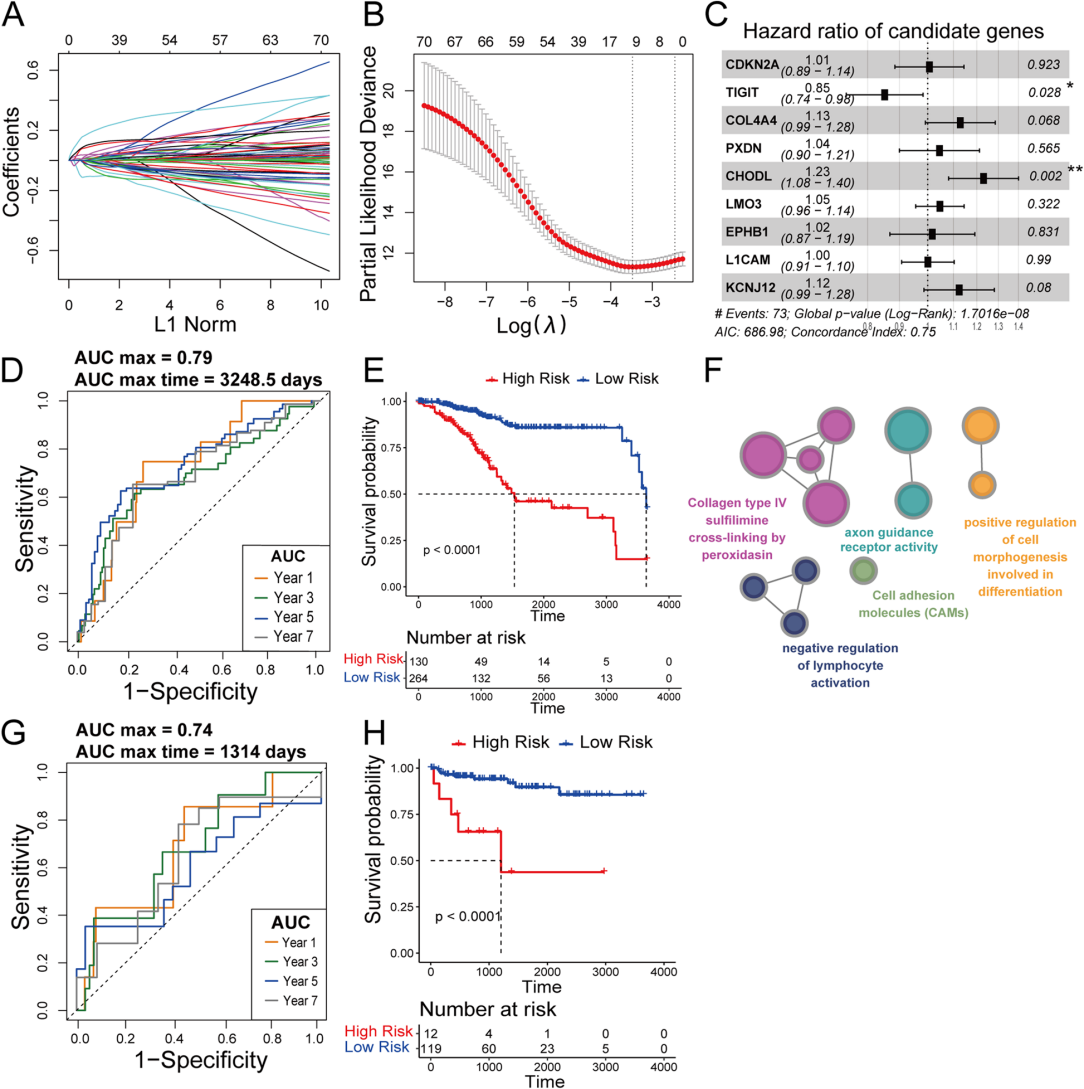
The mRNA signature associated with the m^6^A trait. **A** The profile of the LASSO coefficients. **B** The partial likelihood of deviance is shown against log (Lambda). At the value fitting 10-fold cross-validation, a vertical line was drawn. **C** Forest plots of the prognostic ability of the nine m^6^A-related mRNAs are included in the prognostic signature. Time-dependent ROC curves and Kaplan-Meier survival analysis between UCEC patients with high- and low-risk depend upon our mRNA signature. (**D**, **E**) Training cohort. (**G**, **H**) Testing cohort. We used AUCs at 1, 3, 5, and 7 years to assess prognostic accuracy and calculated *P* values using the log-rank test. **F** Gene Ontology (GO) enrichment of the m^6^A signature

A total of 71 prognostic genes were generated by manipulating a univariate Cox model, revealing an association between the expression level of mRNAs and the OS of patients (*P* < 0.05). To reduce dimensionality and select representative m^6^A-related markers for forecasting the OS of UCEC patients, a LASSO Cox regression model was performed according to the minimum criterion in the training set. Nine m^6^A-related genes, *CDKN2A, TIGIT, COL4A4, PXDN, CHODL, LMO3, KCNJ12, L1CAM, and EPHB1*were selected and further weighted by their regression coefficients (Figs. 4A-B). The forest plot in Fig. 4C shows that *TIGIT* is an independent protective factor with a hazard ratio (HR) < 1 while *CHODL* is an independent risk factor with HR > 1 for UCEC patients (Fig. 4C). The results suggest that patients with a high expression of *CHODL* have a poor survival while those with a high expression of *TIGIT* have a good survival. The whole Cox model also exhibited statistical significance with a concordance index of 0.75.

In addition, time-dependent ROC curve analyses in the training set were performed at 1, 3, 5, and 7 years to evaluate the accuracy of prognostic prediction of the m^6^A-related mRNA signature. Figure 4D shows that the maximum AUC is 0.79 at 3249 days, indicating that m^6^A-related genes can serve as biomarkers in the prognosis of UCEC (Fig. 4D). Subsequently, the risk score for each UCEC patient was calculated with the formula generated by the coefficients. The UCEC patients in the training set were separated into high- and low-risk subgroups using the median risk score as the cut-off value. Consistent with ROC curve analysis, the Kaplan-Meier curve shows that the UCEC patient risk score is inversely proportional to the clinical outcome (Fig. 4E). Figure 4F shows that the gene signature is enriched in the pathways: collagen type IV sulfilimine cross-linking by peroxidasin, axon guidance receptor activity, cell adhesion molecules, positive regulation of cell morphogenesis involved in differentiation, and negative regulation of lymphocyte activation (Fig. 4F).

### Validation of m^6^A gene signature for OS prediction in the testing set

**Fig. 5:**
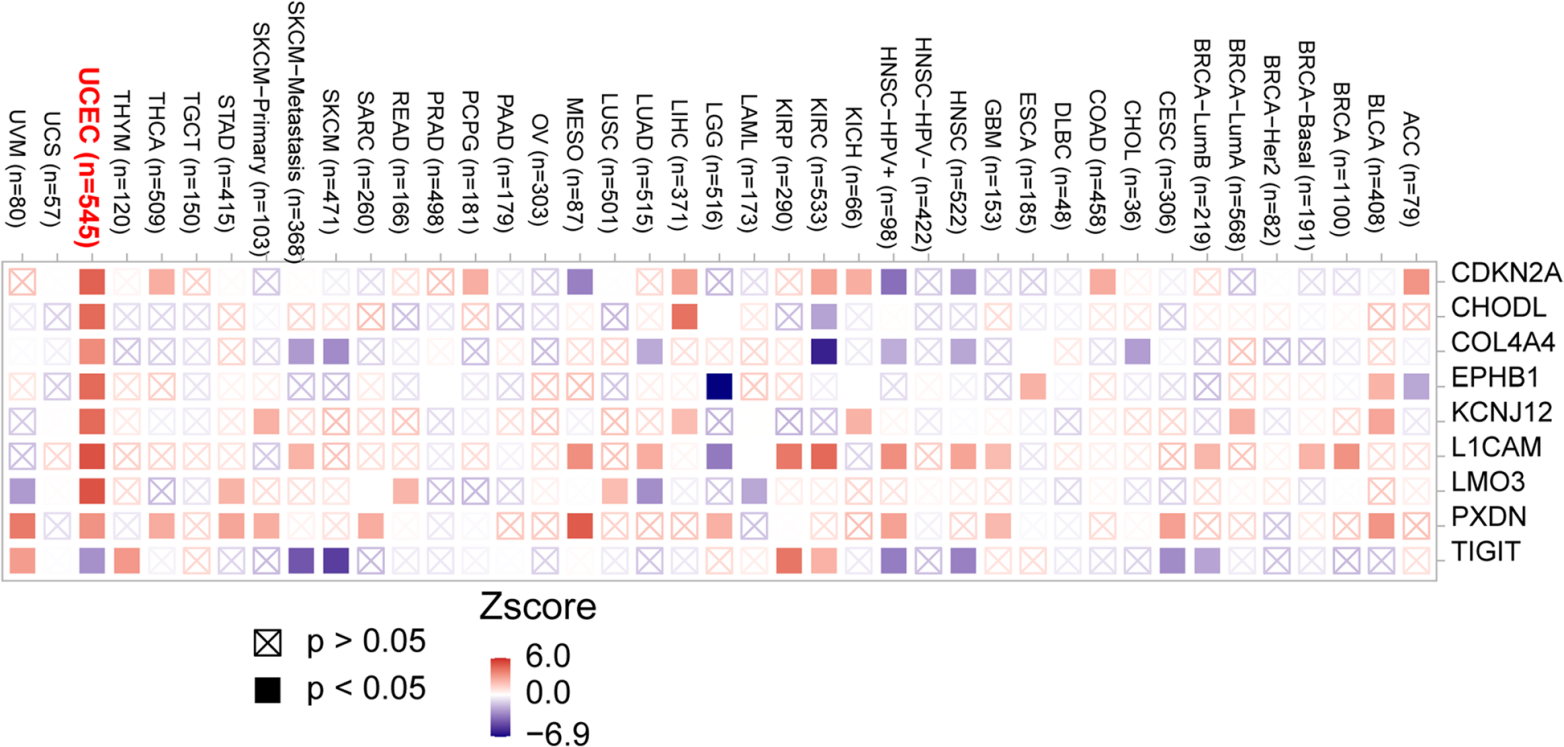
Heatmap shows the relevance of the m^6^A signature gene expression and clinical outcome across various cancer types using the Cox proportional hazard model. The increased risk is marked in red, and the decreased risk is marked in blue

The risk score for each sample in the testing cohort was calculated using the same formula in training set to verify the prognostic ability of the m^6^A gene signature. The UCEC patients in the testing dataset were separated into high- and low-risk groups based on the median risk score. The results were consistent with those in the training dataset. As shown in Fig. 4G, the max AUC is 0.74 at 1314 days (Fig. 4G). Figure 4H shows that patients with higher risk scores have shorter survival and increased death status (*P* < 0.0001), suggesting that the m^6^A gene signature has a robust and stable OS predictive ability (Fig. 4H). Furthermore, Fig. 5 shows that the expression pattern of the m^6^A gene signature is tumor-specific and most closely related to clinical outcomes in UCEC (Fig. 5). The increased risk is marked in red, and the decreased risk is marked in blue. Except for *TIGIT* with a decreased risk, the m^6^A signature has an increased risk for survival. The result is consistent with the finding that abnormal m^6^A levels can lead to tumor genesis [9, 11].

### Further analysis of the proposed m^6^A gene signature

**Fig. 6:**
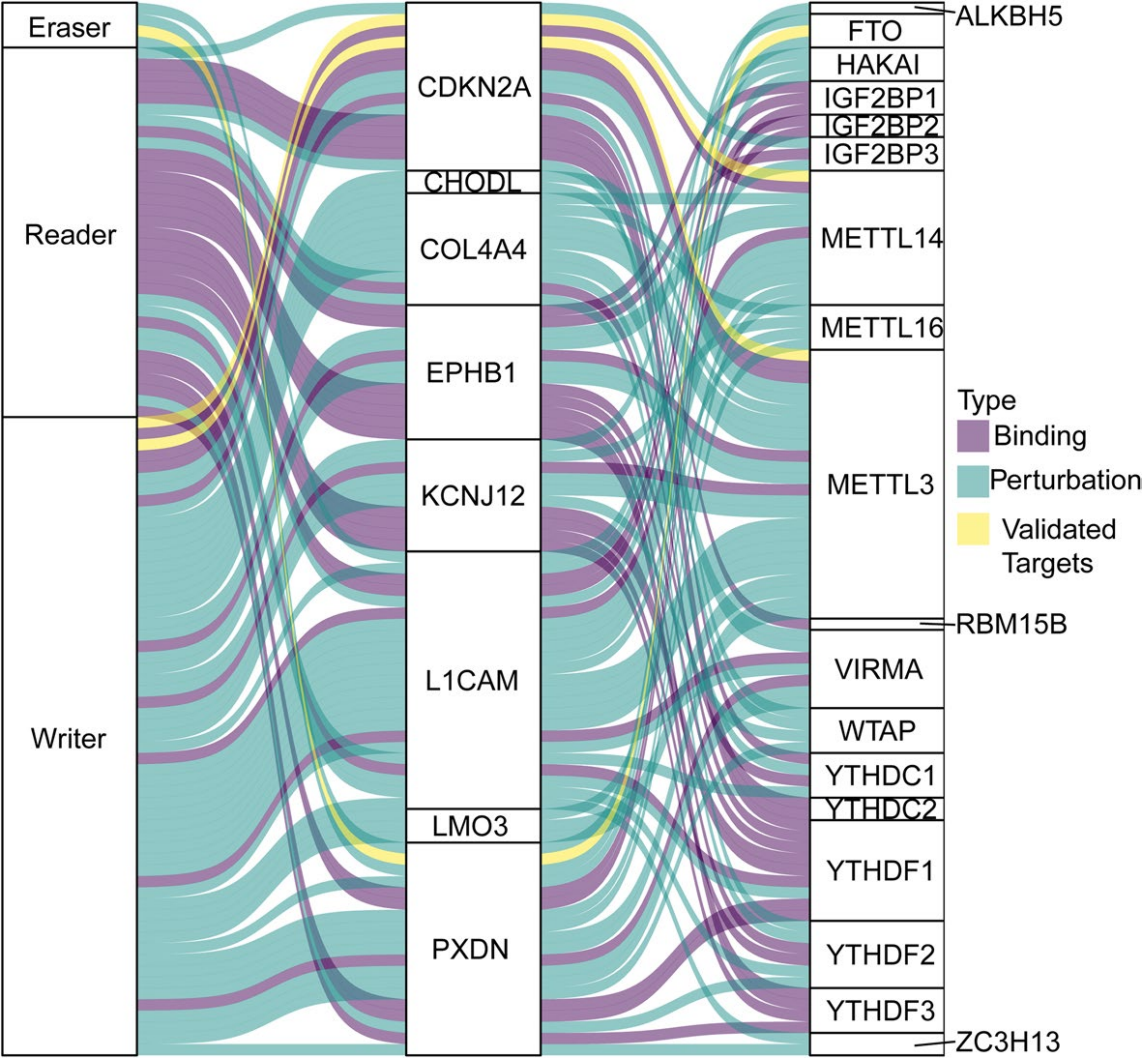
The alluvial plot illustrates the correlation between writers, erasers, and readers (WERs) of m^6^A modification and the m^6^A signature

Figure 6 is the alluvial plot to illustrate the correlation between WERs of m^6^A modification and the m^6^A signature (Fig. 6). Yellow wire represents high confidential targets validated by low-throughput experiments; purple wire represents binding evidence indicated by high-throughput sequencings such as CLIP-Seq, RIP-seq, and ChIP-seq; green wire represents m^6^A WER perturbation followed by high-throughput sequencings such as RNA-Seq, m6A-Seq, and Ribo-Seq [22]. The results show that the proposed m^6^A gene signature closely relates to m^6^A regulators. Therefore, reprogramming m^6^A levels by restoring dysfunction of the m^6^A regulators can directly target the proposed m^6^A gene signature and return to normal expression levels. We explored different RNA modifications other than m^6^A methylation, including N1-methyladenosines (m^1^A), pseudouridine (Ψ) modifications, 5-methylcytosine (m^5^C) modifications, and 2′-O-methylations (2′-O-Me) (Table 3) to better understand the proposed m^6^A signature. The results show that m^6^A methylation is the main type of modification site in the proposed signature.

**Table 3:** RNA Modifications of the Proposed Signature from RMBase v2.0

***Gene***	***Strand***	***m6A Num***	***m1A Num***	***m5C Num***	***2′-O-Me Num***	***PseudoU Num***	***Other Num***	***Total Num***
CDKN2A	–	7	0	0	0	0	0	7
TIGIT	+	1	0	0	0	0	0	1
COL4A4	–	30	0	0	0	0	0	30
PXDN	–	48	0	0	2	0	0	50
CHODL	+	2	0	0	0	0	0	2
LMO3	–	5	0	0	0	0	0	5
KCNJ12	+	25	0	0	0	0	0	25
L1CAM	–	65	0	0	0	0	0	65
EPHB1	+	30	0	0	0	0	0	30

### In vitro and in vivo experiments to validate the proposed m^6^A gene signature

**Fig. 7:**
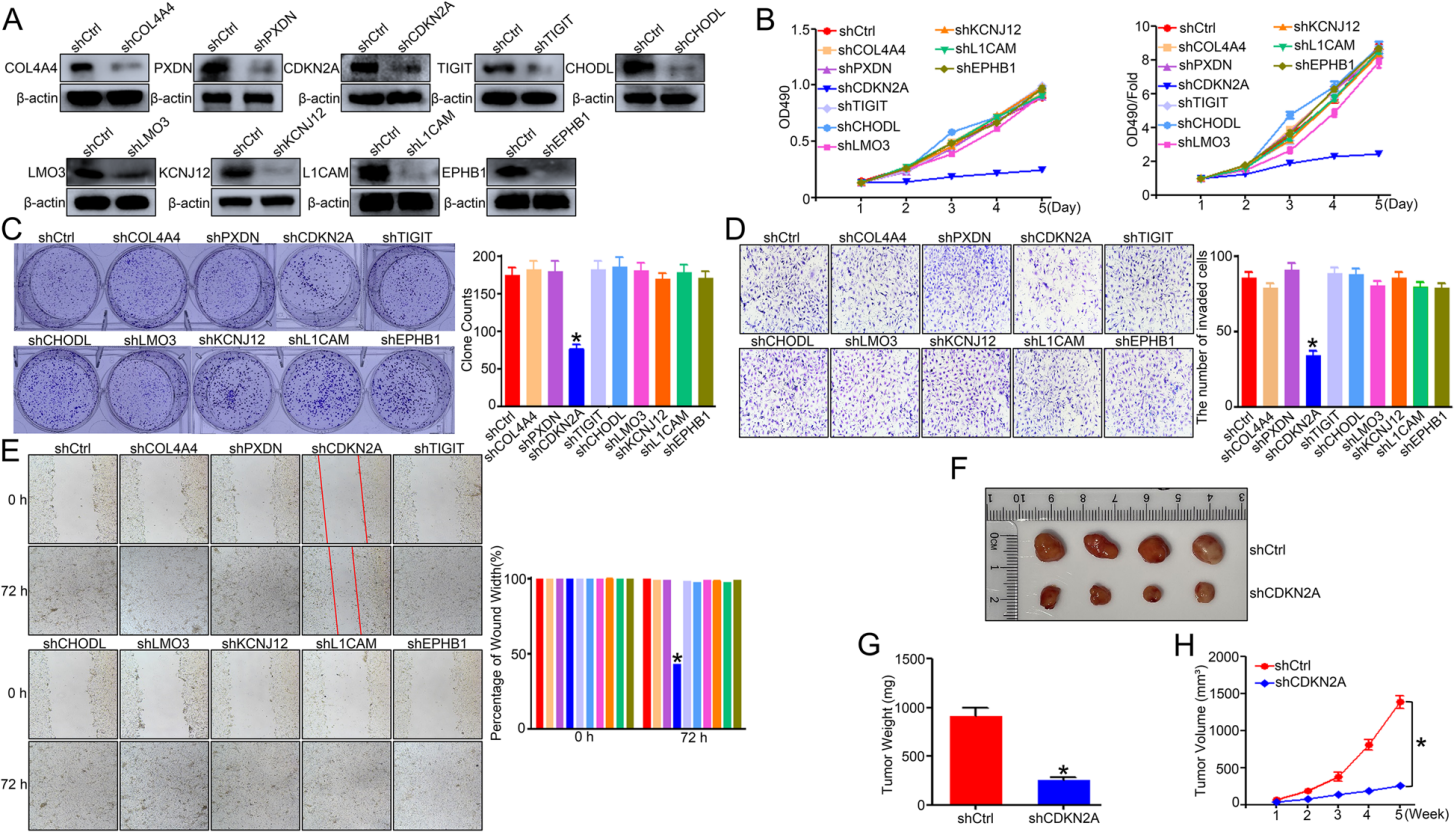
Silencing each gene of the m^6^A signature expression in UCEC inhibits cell proliferation, migration, and invasion in vitro and in vivo*.* **A** Western blot analysis of each gene (*COL4A4, PXDN, CDKN2A, TIGIT, CHODL, LMO3, KCNJ12, L1CAM,* and *EPHB1*) expression in Ishikawa cells expressing each gene shRNA (shCOL4A4, shPXDN, shCDKN2A, shTIGIT, shCHODL, shLMO3, shKCNJ12, shL1CAM, and shEPHB1). β-actin is adopted as a loading control. **B** MTT assays were used to investigate the proliferation rates of the *COL4A4, PXDN, CDKN2A, TIGIT, CHODL, LMO3, KCNJ12, L1CAM, EPHB1*-silenced Ishikawa cells. **C** Colony formation assay was used to investigate the proliferation capacity of the *COL4A4, PXDN, CDKN2A, TIGIT, CHODL, LMO3, KCNJ12, L1CAM, EPHB1*-silenced Ishikawa cells. Representative pictures are shown on the left, and the number of colonies has been counted on the right. **D** Transwell assay was used to investigate the metastasis capacity of the *COL4A4, PXDN, CDKN2A, TIGIT, CHODL, LMO3, KCNJ12, L1CAM, EPHB1*-silenced Ishikawa cells. Representative pictures are shown on the left, and the number of metastasis cells has been counted on the right. **E** Wound healing assay was used to investigate the migration capacity of the *COL4A4, PXDN, CDKN2A, TIGIT, CHODL, LMO3, KCNJ12, L1CAM, EPHB1*-silenced Ishikawa cells. Representative pictures are shown on the left, and the wound width has been measured on the right. **F** Macroscopic view of tumor harvested of two indicates treatment groups (*n* = 4). **G** Comparison of the tumor weight of two indicated treatment groups at the end-point (*n* = 4). **H** Comparison of the growth curve of the tumors of two indicates treatment groups at the each-point (*n* = 4). Bar graph data are presented as mean ± SEM; *, *P* < 0.05

Then, some in vitro experiments were performed to verify the above assumption. The Ishikawa cell line is by far the most commonly used xenograft model of UCEC. To elucidate the biological functions of *CDKN2A, TIGIT, COL4A4, PXDN, CHODL, LMO3, KCNJ12, L1CAM,* and *EPHB1* in UCEC tumor cells, we knocked down the expression of the 9-gene signature using shRNA or the negative control in Ishikawa cell lines to assess cell proliferation, migration, and invasion in vitro (Fig. 7A). The MTT assay and the colony formation assay demonstrated that tumor proliferation was significantly inhibited in the shCDKN2A group compared with the shCtrl group (*P* < 0.05, Figs. 7B-C). Transwell assays revealed that Ishikawa cell invasion was significantly reduced after downregulation of *CDKN2A* (Fig. 7D). Cell migration was evaluated by wound-healing assay. The result shows that decreased expression of *CDKN2A* significantly inhibits the migration of Ishikawa cells (Fig. 7E). The above results indicate that knockdown of *CDKN2A* can inhibit the proliferation, migration, and invasion of UCEC tumor cells in vitro. The mouse xenograft models were established to determine whether *CDKN2A* contributes to tumor progression in vivo. The shRNA or *CDKN2A* shRNA Ishikawa cells were injected at flank into nude mice. After 5 weeks of injection, the tumor volume and weight of the *CDKN2A* knockdown group were significantly reduced compared to those of the control group (Figs. 7F-H). These findings indicated that *CDKN2A* functions from the perspective of tumor cells since it is significantly up-regulated in metastatic UCEC, and its knockdown attenuates the ability of tumor cells to invade metastases in vitro and in vivo.

### Potential immunological role of the m^6^A gene signature

**Fig. 8:**
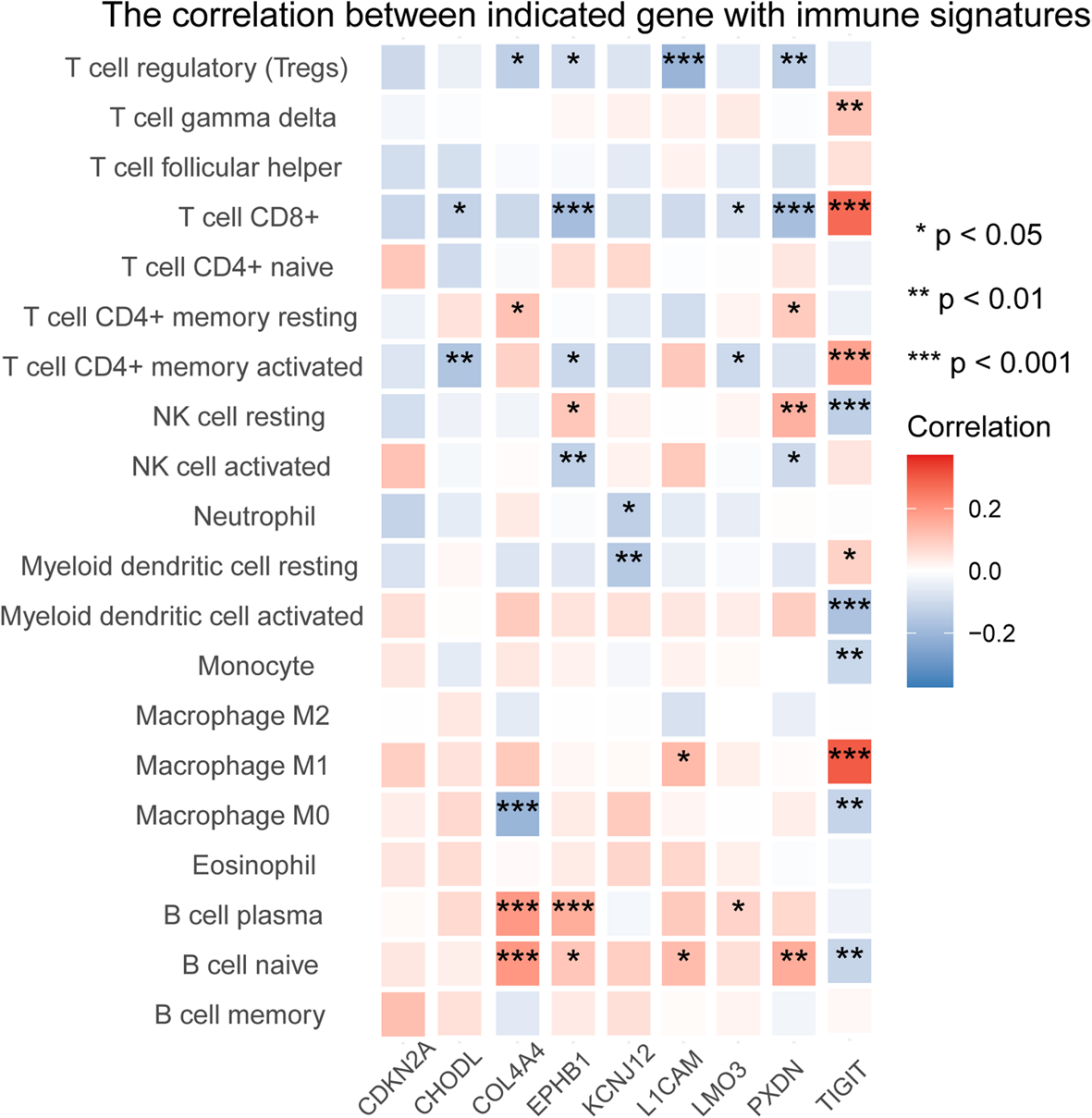
Correlation between each gene of the m^6^A gene signature and the signatures of various types of immune cells

Since only *CDKN2A* among all 9 genes was validated to be associated with tumor cells in in vitro and in vivo assays, we speculated that the rest of the genes function from the perspective of immune cells. The correlation between various types of immune cell signatures and each gene of the m^6^A gene signature was explored (Fig. 8). Using the CIBERSORT algorithm, we found that except for *CDKN2A*, other genes had a certain degree of correlation with immune cells. *TIGIT* was the gene most strongly associated with immune function and was associated with CD8^+^ T cells, CD4^+^ T cells, NK cells, and Macrophage M1 cells with *P* < 0.001. In contrast, its trend was the opposite for other genes. The result is consistent with the previous results: *TIGIT* is an independent protective factor with a hazard ratio (HR) < 1 while others are risk factors. These results indicate that *CDKN2A* may function from the perspective of tumor cells, and *CDKN2A, TIGIT, COL4A4, PXDN, CHODL, LMO3, KCNJ12, L1CAM, and EPHB1* may play a role in UCEC from an immunological point of view.

## Discussion

UCEC is the most common female pelvic malignancy worldwide. The m^6^A modification, a dynamic and reversible process involving m^6^A regulators, can directly regulate the expression of oncogenes or tumor suppressor genes at the transcriptional level [8], which is closely associated with malignancies. A better understanding of the potential role of m^6^A modification during the tumorigenesis can provide potential biomarkers for early diagnosis and prognosis assessment and new ideas for therapeutic targets [24, 25]. Abnormal expression patterns of m^6^A regulators and a reduction in m^6^A methylation have already been found in endometrial cancer [11]. However, the genetic variations, tumor-infiltrating immunological characteristics, and clinical implications involving m^6^A regulators in UCEC remain unknown.

In the present study, we examined the landscape of genetic alterations in the m^6^A regulators of TCGA UCEC patients and found that 28.9% of m^6^A regulators were mutated, suggesting a close relationship between m^6^A methylation modification and endometrial cancer. Among them, the highest frequency of genetic alteration was found in the m^6^A writer gene *ZC3H13* (11.9%). The result shows that the function of *ZC3H13* is suppressed in UCEC patients, and this dysfunction may be caused by the occurrence of UCEC. *ZC3H13* has also been reported to act as a tumor suppressor in breast and colorectal cancer [26, 27]. Then, we explored the relevance of genetic alterations, m^6^A gene expression, and the clinical phenotypes of patients with UCEC. Most indicators did not differ significantly between two distinct traits of m^6^A regulator alteration, except that UCEC patients younger than 60 are more likely to have genetic alterations of m^6^A regulators. The result implies that although older patients are more likely to be informed of the risks and symptoms of endometrial cancer, m^6^A modifications may play a role in the occurrence of endometrial cancer in younger patients [28]. Alteration in only one m^6^A regulator can lead to the dysfunction of m^6^A RNA modification. Therefore, we generated an mRNA signature from the outcome-related DEGs by univariate Cox regression followed by LASSO Cox regression after the separation of UCEC patients into two subgroups depending upon the distinction of the m^6^A regulator. Subsequently, a nine m^6^A-related prognostic signature, *CDKN2A, TIGIT, COL4A4, PXDN, CHODL, LMO3, KCNJ12, L1CAM, and EPHB1*, was developed and performed well for predicting the survival outcome of UCEC patients.

To better understand the proposed m^6^A signature, we explored different RNA modifications other than m^6^A methylation and illustrated the correlation between m^6^A regulators and the proposed signature. The results show that the proposed m^6^A gene signature did have a close relationship with m^6^A regulators. Furthermore, m^6^A methylation is the main modification site in the proposed signature since the direct effects of m^6^A modification on modified transcript expression are established, and the epigenetic reprogramming is more likely manipulated by targeting epigenetic modifiers [29]. Therefore, this m^6^A signature can provide new ideas for diagnosing and treating UCEC from the perspective of epigenetics.

Since RNA-seq data in TCGA is performed using bulk RNA extracted from homogenized tissues, which provide an average number of gene expressions in the pooled population of diverse cells [30], we cannot determine whether the proposed gene signature works in tumor cells or tumor-infiltrating immune cells. Therefore, we used in vitro and in vivo tumor cell experiments and immune correlation analysis to verify the function of each gene in the proposed gene signature. The results show that *CDKN2A* mainly acts from the perspective of tumor cells, while *COL4A4, PXDN, TIGIT, CHODL, LMO3, KCNJ12, L1CAM, and EPHB1* may play a role in UCEC from an immunological point of view. Numerous studies have demonstrated that m^6^A regulators correlate with malignant tumor progression and immunomodulatory abnormality, revealing the special correlation between TME infiltrating immune cells and m^6^A modification [12]. A recent study also shows that abnormal modification behavior of m^6^A can slow down the kinetic rates of mRNA during T cell differentiation due to the increase in naïve T cell numbers from lymph nodes [31]. Therefore, our proposed prognostic m^6^A gene signature may predict immune therapeutic efficacy. Since the present study focused on mining the m^6^A associated gene signature and defined their roles from tumor and immune perspective separately without the mechanistic studies that shows the downstream targets of each gene. We will use our constructed knockdown cell lines for RNA-seq to obtain gene expression profiles of KD cell lines relative to control cell lines that have been subjected to subsequent mechanistic studies.

## Conclusions

The abnormal m^6^A modifications are closely associated with the clinical outcome of patients with UCEC. The m^6^A RNA methylation regulator-based prognostic signature can predict the prognosis of UCEC patients and immune therapeutic efficacy from the perspective of epigenetics. Our results will provide a direction for the further exploration of the pathogenesis of UCEC.

## Supplementary Information

**Additional file 1: Supplementary Fig. 1** m^6^A gene expression between UCEC patients with or without mutations in m^6^A regulators. *, *P* < 0.05; **, *P* < 0.01.

## Data Availability

Publicly available datasets were analyzed in this study. These datasets can be found here: https://portal.gdc.cancer.gov/.

## Abbreviations

*UCEC*: Uterine corpus endometrial carcinoma
*m*^*6*^*A*: N6-methyladenosine
*TCGA*: The Cancer Genome Atlas
*OS*: Overall survival
*LASSO*: Least absolute shrinkage and selection operator
*ROC*: Receiver operating characteristic
*m*^*1*^*A*: N^1^-methyladenosine
*m*^*5*^*C*: 5-methylcytosine
*WERs*: Writers, erasers, and readers
*TME*: Tumor microenvironment
*APC*: Antigen-presenting cells
*MAF*: Mutation annotation format
*HGNC*: HUGO Gene Nomenclature Committee
*DEGs*: Differentially expressed genes
*GO*: Gene ontology
*AUC*: Area under the ROC curve
*Ψ*: Pseudouridine
*2′-O-Me*: 2′-O-methylations
