# Supplementary material for: m6A RNA methylation regulator-based signature for prognostic prediction and its potential immunological role in uterine corpus endometrial carcinoma
Source: BMC Cancer. 2022 Dec 29;22:1364. doi: 10.1186/s12885-022-10490-x (PMC9801604; doi:10.1186/s12885-022-10490-x)
Supplement: Supplementary file 1 — Additional file 1: Supplementary Fig. 1 m6A gene expression between UCEC patients with or without mutations in m6A regulators. *, P < 0.05; **, P < 0.01. [file 12885_2022_10490_MOESM1_ESM.docx]

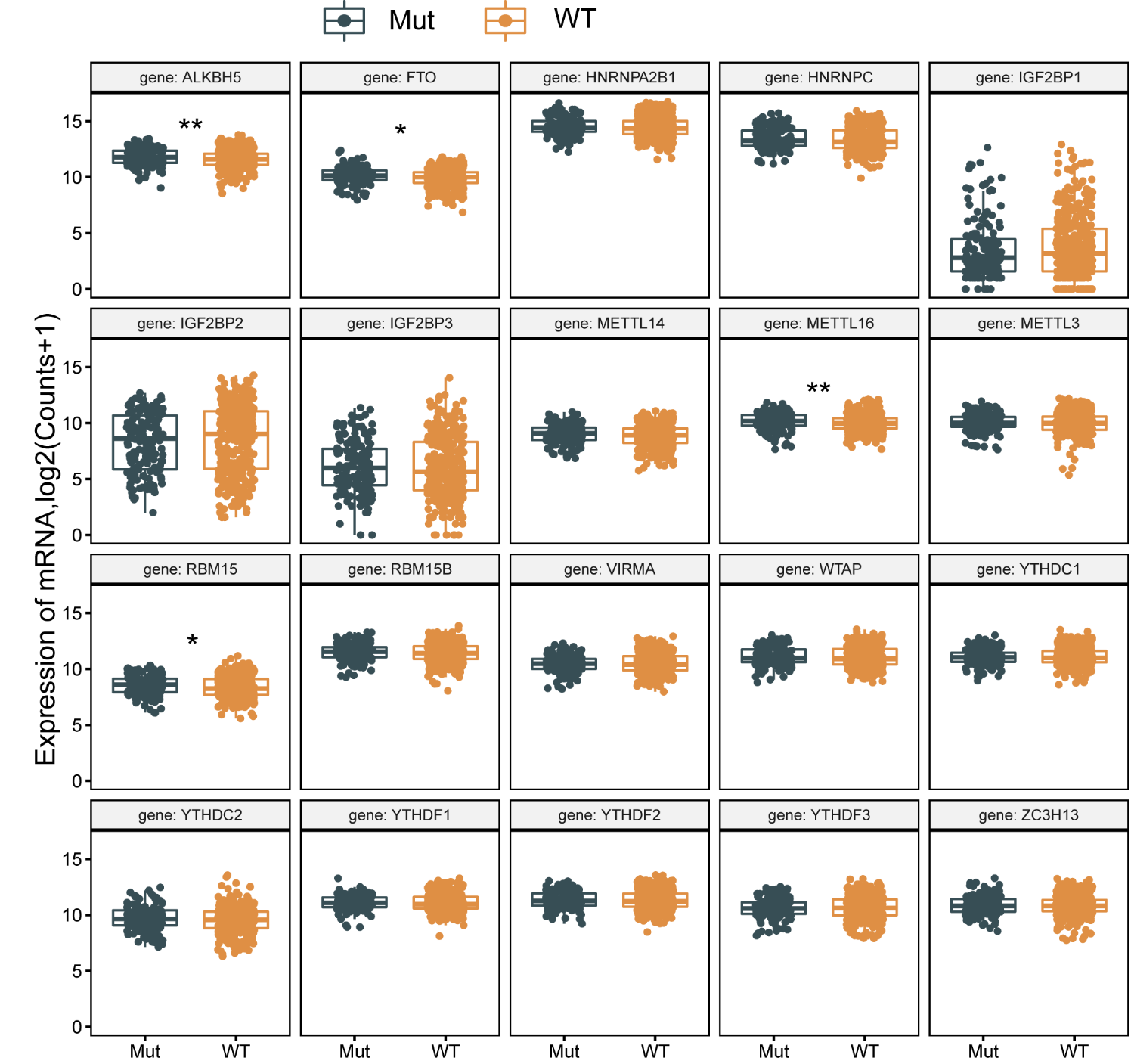


**Supplementary Figure 1** m^6^A gene expression between UCEC patients with or without mutations in m^6^A regulators. *, *P* < 0.05; **, *P* < 0.01.
